# Antileishmanial Activity of Medicinal Plants Used in Endemic Areas in Northeastern Brazil

**DOI:** 10.1155/2014/478290

**Published:** 2014-07-13

**Authors:** Aline Cavalcanti De Queiroz, Thays de Lima Matos Freire Dias, Carolina Barbosa Brito Da Matta, Luiz Henrique Agra Cavalcante Silva, João Xavier de Araújo-Júnior, Givanildo Bernardino de Araújo, Flávia de Barros Prado Moura, Magna Suzana Alexandre-Moreira

**Affiliations:** ^1^Laboratório de Farmacologia e Imunidade (LaFI), Instituto de Ciências Biológicas e da Saúde, Universidade Federal de Alagoas, 57072-970 Maceió, AL, Brazil; ^2^Laboratório de Pesquisa em Recursos Naturais, Universidade Federal de Alagoas, Maceió, AL, Brazil; ^3^Laboratório de Plantas Tropicais (LPT), PPG-Dibict, Instituto de Ciências Biológicas e da Saúde, Universidade Federal de Alagoas, Maceió, AL, Brazil

## Abstract

This study investigates the leishmanicidal activity of five species of plants used in folk medicine in endemic areas of the state of Alagoas, Brazil. Data were collected in the cities of Colonia Leopoldina, Novo Lino, and União dos Palmares, Alagoas state, from patients with cutaneous leishmaniasis (*Leishmania amazonensis*) who use medicinal plants to treat this disease. Plants extracts were tested at a concentration of 1–100 *μ*g/mL in all experiments, except in an assay to evaluate activity against amastigotes, when 10 *μ*g/mL was used. All plants extracts did not show deleterious activity to the host cell evidenced by LDH assay at 100, 10, and 1 *μ*g/mL after 48 h of incubation. The plants extracts *Hyptis pectinata* (L.) Poit, *Aloe vera* L., *Ruta graveolens* L., *Pfaffia glomerata* (Spreng.) Pedersen, and *Chenopodium ambrosioides* L. exhibited direct activity against extracellular forms at 100 *μ*g/mL; these extracts inhibited growth by 81.9%, 82.9%, 74.4%, 88.7%, and 87.4%, respectively, when compared with promastigotes. The plants extracts *H. pectinata, A. vera,* and *R. graveolens* also significantly diminished the number of amastigotes at 10 *μ*g/mL, inhibiting growth by 85.0%, 40.4%, 94.2%, and 97.4%, respectively, when compared with control. Based on these data, we conclude that the five plants exhibited considerable leishmanicidal activity.

## 1. Introduction

The Trypanosomatidae comprise a large group of parasitic protozoa, some of which cause important diseases in humans [1]. For example, leishmaniasis is an infectious disease that is transmitted by insects and is prevalent in Europe, Africa, Asia, and the Americas. It causes significant morbidity and mortality and thus constitutes a serious public health problem [2]. Each year, the parasite kills thousands and debilitates millions of people; 2 million new cases are reported annually, and 350 million people are at risk [3]. Leishmaniasis is endemic in 98 countries [4], 82% of which are low-income countries.

*Leishmania* is an obligatory intracellular parasite of monocytes and macrophages and has a digenetic life cycle that alternates between two stages: flagellated promastigotes, which develop in the midgut of the insect vector, and amastigotes, which multiply in the host macrophage [5, 6]. The disease can be caused by nearly 21* Leishmania* species and encompasses a spectrum of clinical manifestations, including cutaneous lesions, oropharyngeal mucosa inflammation, and visceral infection [1]. Thus, leishmaniasis can be categorized broadly into three types: (i) cutaneous leishmaniasis, in which parasites remain at the site of infection and cause localized long-term ulceration, (ii) mucocutaneous leishmaniasis, a chronic destruction of mucosal tissue that develops from the cutaneous disease in less than 5% of affected individuals; and (iii) visceral leishmaniasis, the most serious form, in which parasites leave the inoculation site and proliferate in liver, spleen, and bone marrow, resulting in host immunosuppression and ultimately death in the absence of treatment [3].

Tegumentary leishmaniasis in New World is referred to as American tegumentary leishmaniasis (ATL) a serious zoonosis specially endemic in a lot of areas of Latin America. It is caused by* Leishmania* species of the subgenera* Leishmania *(*Viannia*) and (*Leishmania*) and is distributed from the south of the United States to the north of Argentina [2, 7]. In Brazil, ATL is detected in all states and has shown a high incidence over the last 20 years. There is wide genetic diversity among the* Leishmania* parasites; at least seven* Leishmania* species have been described as the etiological agent of human cutaneous disease, with most cases being caused by* Leishmania* (*Viannia*)* braziliensis*. However,* L. amazonensis* is also important as a causative agent of ATL, having been reported in the northeast, southeast, and west-central parts of Brazil [7–9].

Unfortunately, antileishmanial drugs, which are mainly based on antimonial therapy, are toxic, and recently developed and tested vaccines have shown relatively low protection under field conditions [6]. Pentavalent antimony (SbV) compounds such as sodium stibogluconate and meglumine antimoniate are the first choice of therapy for leishmaniasis. Despite their extensive clinical use for several decades, the mechanism of action remains unclear [10]. Other drugs used to treat leishmaniasis include pentamidine and amphotericin B, but the use of these drugs has been limited due to their high toxicity and cost [11]. Recently, the oral drug miltefosine was approved for the treatment of human visceral* Leishmania* infections, and fluconazole taken orally is also effective against cutaneous leishmaniasis [12].

Because of the adverse side effects of these treatment regimens, considerable attention has been given to the discovery and development of new, less toxic chemotherapeutic agents [13]. In an ongoing search for improved and cheaper leishmanicidal agents, plant-derived products represent an attractive option. According to the World Health Organization (WHO), approximately 80% of the world's inhabitants rely on traditional medicines for their health care [14]. The discovery of artemisinin, a sesquiterpene lactone produced by* Artemisia annua*, as a pharmaceutical for the treatment of malaria promoted interest in the discovery of new compounds from plants with antiprotozoal activity, especially those plants used against* Leishmania* parasites [15].

Because it is a very common disease in the Brazilian northeast leishmanioses has been traditionally treated with folk medicine using native and cultivated plants. Currently, even with access to conventional treatments, patients continue to use plants that are seen by them as having the capacity to heal sores resulting from cutaneous leishmaniasis. This study investigated the leishmanicidal activity against* Leishmania amazonensis* of five species of plants,* Ruta graveolens* L.,* Aloe vera* L.,* Chenopodium ambrosioides* L.,* Pfaffia glomerata* (Spreng.) Pedersen, and* Hyptis pectinata* (L.) Poit, popularly known as arruda, babosa, mastruz, meracilina, and sambacaitá, respectively, used in folk medicine in endemic areas of the state of Alagoas, Brazil, to treat cutaneous leishmaniasis [16]. These plants also have broad medicinal use on the part of other traditional peoples in the Brazilian northeastern region [17].

## 2. Materials and Methods

### 2.1. Ethnobotanical Survey and Plant Collection

The five species of plants examined are broadly used in Brazilian folk medicine for various purposes, including folk therapy to treat leishmaniasis. These species were selected based on the popular use of plants to treat this disease in Alagoas state. The ethnobotanical research was carried out after the community members were fully informed of its purpose. Aerial parts of* H. pectinata (MUFAL 4050)*,* A. vera (MUFAL 4052), *and* R. graveolens (MUFAL 4051)* were collected in the city of Novo Lino. Aerial parts of* P. glomerata (MUFAL 4053)* were collected in the city of Colônia Leopoldina.* C. ambrosioides (MUFAL 4054) *was collected in the city of União dos Palmares. The samples were identified in Natural History Museum/UFAL.

### 2.2. Preparation of Aqueous Extracts

Aerial parts of* H. pectinata*,* R. graveolens*,* P. glomerata,* and* C. ambrosioides* were dried in an oven at 40°C for 96 h, pulverized, and processed with watch by infusing. Fresh succulent leaves of* A. vera* were crushed in an electric grinder, and the resultant slurry was used as the aqueous extract from this plant. The solutions were filtered and sterilized by filtering through sterile 0.22 *μ*m membranes. For the experiments, the dry weight of each aqueous extract per mL was measured to determine the amount of solution required to achieve a given concentration in each well.

### 2.3. Biological Assays

#### 2.3.1. Parasite Culture

*L. amazonensis* (IFLA/BR/1967/PH8 strain) promastigotes were grown in culture medium (Schneider's medium (Gibco, Life Technologies, São Paulo, Brazil)) containing 10% fetal bovine serum (FBS, Gibco, Life Technologies, São Paulo, Brazil), 2% human urine, and gentamicin (10 *μ*g/mL) at 27°C in a BOD incubator. This strain was kindly provided by the* Leishmania* collection of the Oswaldo Cruz Institute, Rio de Janeiro, Brazil.

#### 2.3.2. Culture of J774 Murine Macrophages

This adherent-phenotype macrophage line was cultured in Dulbecco's Modified Eagle's medium (DMEM, Sigma, São Paulo, Brazil) with 10% FBS and gentamicin (10 *μ*g/mL) at 37°C under 95% humidity and 5% CO_2_.

#### 2.3.3. *In Vitro* Cytotoxicity Assays

The deleterious effects of the aqueous extracts of* H. pectinata, A. vera*,* R. graveolens*,* P. glomerata, *and* C. ambrosioides* were determined by assessing their cytotoxicity on murine macrophages (J774 cell line). Briefly, cell suspensions containing 7.5 × 10^5^ cells/mL were placed in a 96-well plate in triplicate and incubated at 37°C for 1 h. Then, each aqueous extract was added at three serial dilutions starting at 100 *μ*g/mL (final volume: 200 *μ*L; concentrations: 100, 10, and 1 *μ*g/mL). Cell growth medium free from aqueous extracts was used as a basal growth control. Pentamidine was used as a drug standard and was added at three serial dilutions: 100, 10, and 1 *μ*M. Cytotoxicity that was unrelated to pentamidine was assessed using the solvent DMSO (Sigma) as a vehicle control in this assay (DMSO was used to solubilize the pentamidine). For the vehicle control, the volume used to dissolve the pentamidine was considered as the highest concentration and also serially diluted. After cells had been exposed to plant aqueous extract, pentamidine or DMSO for 48 h at 37°C and 5% CO_2_, the assay was performed in an absorbance microplate reader to measure lactate dehydrogenase (Doles) [18].

#### 2.3.4. Antileishmanial Assay against* L. amazonensis*

The Ethics Committee of Federal University of Alagoas (No. 014869/2006-86) approved all experimental protocols described in this study.

The cytotoxic effects of the aqueous extracts of* H. pectinata*,* A. vera*,* R. graveolens*,* P. glomerata,* and* C. ambrosioides* against promastigote forms were determined. Stationary phase* L. amazonensis* promastigotes were plated in 48-well vessels (Nunc) at 1 × 10^6^ cells per well in Schneider's medium, supplemented with 10% FBS, 2% human urine, and gentamicin (10 *μ*g/mL). The aqueous extracts were added at three serial dilutions (100, 10 and 1 *μ*g/mL), and cell growth medium free from aqueous extracts was used as a basal growth control. Pentamidine was used as the drug standard and was added at three serial dilutions: 100, 10, and 1 *μ*M. After 3 d, the extracellular load of* L. amazonensis* promastigotes was estimated by Neubauer chamber counting of the number of extracellular motile promastigotes in Schneider's medium [19].

The aqueous extracts of* P. glomerata* and* C. ambrosioides* were only evaluated in the experiments measuring cytotoxicity against promastigotes and macrophages because of the low amount of plant material obtained. Because the aim of this work was to mimic the popular use of plants commonly used in endemic areas of Alagoas (Brazil), we only obtained plants from the places in which the populace obtains them.

To evaluate the antileishmanial activity of the plants against extracellular replication, murine macrophages (the J774 cell line) were plated in 48-well vessels at 1.5 ×10^5^ cells per well in complete culture medium. The cells immediately received 1 × 10^6^ stationary phase* L. amazonensis* promastigotes and were incubated in complete medium supplemented with 10% FBS at 37°C. After 4 h, monolayers were extensively washed with warm HBSS (Sigma) to remove extracellular parasites and nonadherent cells, leaving approximately 1 × 10^5^ adherent macrophages. All cultures were grown in DMEM supplemented with 10% FBS. The aqueous extracts were added at three serial dilutions from 100 to 1 *μ*g/mL, and cell growth medium free from aqueous extracts was used as a basal growth control. Pentamidine was used as a drug standard and was added at three serial dilutions (100–1 *μ*M). Extracellular parasites were absent throughout this period. After 3 d, the infected macrophage monolayers were extensively washed, and the medium was replaced by 0.5 mL Schneider's medium, supplemented with 10% FBS and 2% human urine [19]. The monolayers were cultured at 26°C for an additional 3 d. The intracellular load of* L. amazonensis* amastigotes was estimated based on the production of proliferating extracellular motile promastigotes in Schneider's medium [20, 21].

To assess the activity of the plant aqueous extracts against the amastigote stage of the parasites, we utilized a model of infection on a cover glass [22]. The murine macrophages (the J774 cell line) were prepared in 24-well vessels (Corning) at 2 × 10^5^ adherent cells/well and infected with 2 × 10^6^ promastigotes in glass coverslips, inside which was placed 1 mL of culture medium. The cultures were cultured with or without the plant aqueous extracts (10 *μ*g/mL) for 3 d under 37°C, 7% CO_2_. After 3 d, the coverslips were washed and stained using the Panoptic staining kit (LB), and the numbers of intracellular amastigotes were counted per 100 macrophages. The results are presented as the number of amastigotes per 100 macrophages and as a percentage of the infected macrophages.

### 2.4. Statistical Analysis

Data obtained from the* in vitro *experiments are expressed as the mean ± standard error of the mean (mean ± S.E.M.) of triplicate cultures. Significant differences between treated and control groups were evaluated using ANOVA and Dunnett post hoc tests. Differences with a *P* value of <0.05 were considered significant.

## 3. Results and Discussion

**Figure 1:**
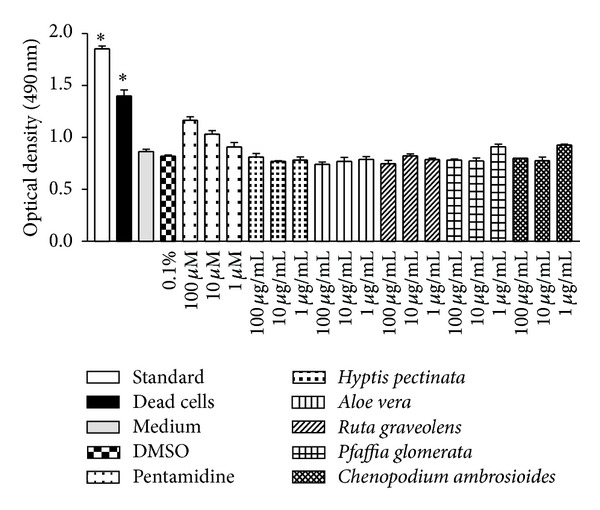
Cytotoxicity of* H. pectinata*,* A. vera*,* R. graveolens*,* P. glomerata,* and* C. ambrosioides* in mammalian cells. The experiments were performed three times independently, and each count was performed in triplicate. The data are reported as the means ± S.E.M. Differences with *P* values <0.05 were considered significant.

Determining the absence of toxic effects on host cells is an important criterion that must be evaluated when searching for active compounds with therapeutic potential against* L. amazonensis*. Results show the degree of toxicity of* C. ambrosioides*,* H. pectinata*,* R. graveolens*,* A. vera,* and* P. glomerata* (and pentamidine, the positive control) as measured in the J774 murine macrophage model (Figure 1). All plants used in this study exhibited no activity that was deleterious to the host cell, as evident from the viability of macrophages treated with 100, 10, and 1 *μ*g/mL after 48 h of incubation, whereas LDH values of cultures treated with plant extracts were similar to control treated with media only or vehicle. This finding is very important for* A. vera*, in particular, because its leaf exudate contains saponin, which is toxic to monocytes. In a previous* in vitro* study, which was performed using murine peritoneal macrophages and a human monocyte cell line U937 that had been exposed to* A. vera* leaf exudate (300 *μ*g/mL), minimal cell death was observed [23], in agreement with the present work. Moreover, the treatment with pentamidine did not show cytotoxic effect to host cells; however this standard drug at 100 *μ*M presented optical density 25% greater than the vehicle group (DMSO 0.1%).

Furthermore, the determination of plants cytotoxicity against host cells is important in order to determine the selectivity of these against* Leishmania*, in particular, against amastigotes, as these are the evolutionary forms found in the mammalian hosts. Therefore, it is important that the plants have toxicity against* Leishmania* and not against the host cell. Moreover, this test is important to determine the concentrations that can be used to evaluate activity against intracellular amastigotes, since macrophages are used as host cells in the assay.

**Figure 2:**
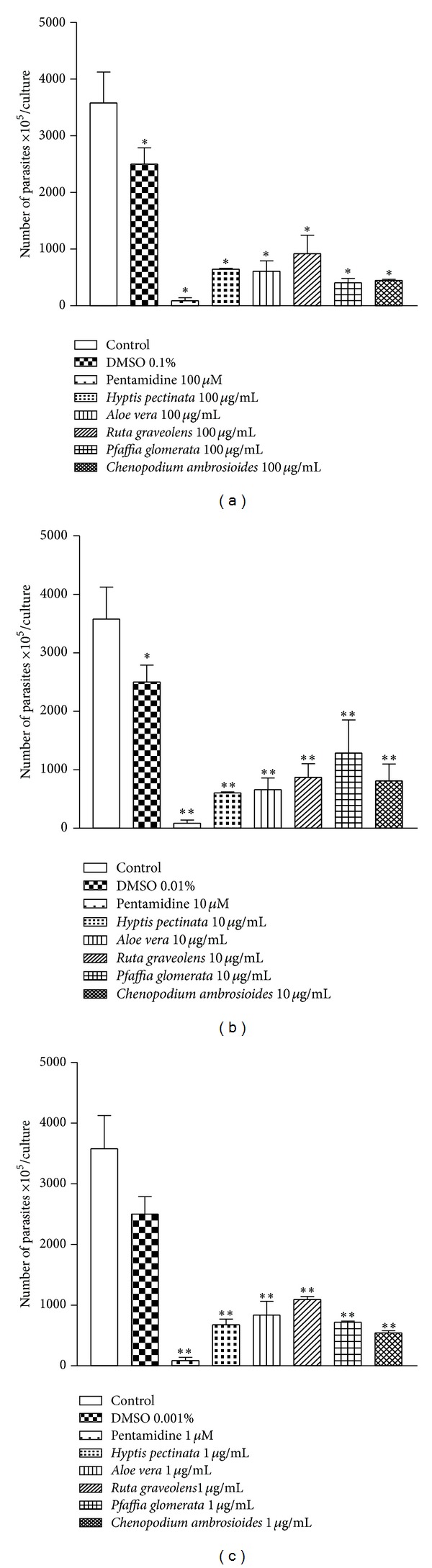
Direct antileishmanial effect of* H. pectinata*,* A. vera*,* R. graveolens*,* P. glomerata,* and* C. ambrosioides* against* L. amazonensis* promastigotes. The height of the bars indicates the number of parasites at each concentration of plant aqueous extract compared with the control experiment containing only the solvent DMSO. The plant aqueous extracts were added at concentrations of 100, 10, and 1 *μ*g/mL in cultures of* L. amazonensis* promastigotes for 4 days. After 4 days, the extracellular load of* L. amazonensis* was measured. The experiments were performed three times independently, and each count was performed in triplicate. The data are reported as the means ± S.E.M. Differences with *P* values <0.05 were considered significant (*P < 0.05; **P < 0.01).

The activities of the aqueous plant extracts against the extracellular replication of* L. amazonensis* were evaluated using* in vitro* assays (Figure 2), “which indirectly measure the effect against intracellular forms of* Leishmania*.”* H. pectinata*,* A. vera*,* R. graveolens*,* P. glomerata,* and* C. ambrosioides* exhibited direct activity against extracellular forms; the observed percentages of growth inhibition were 74.2%, 75.6%, 70.8%, 83.8%, and 82.1%, respectively, at 100 *μ*g/mL, but were less potent than pentamidine (with maximum effect of 96.5% at 100 *μ*M).

*L. amazonensis* is of particular importance because it is one of the most frequent species that causes human disease and specifically causes anergic diffuse leishmaniasis, a disfiguring cutaneous disease that is very difficult to cure [24]. In this study,* H. pectinata*,* A. vera*,* R. graveolens*,* P. glomerata, and C. ambrosioides *were shown to be active against promastigotes, and* H. pectinata*,* A. vera*, and* R. graveolens* were shown to be active againstintracellular amastigotes of* L. amazonensis.* In addition, pentamidine was the most effective compound against promastigote and amastigote forms of* L. amazonensis*, presenting efficacy of 98.9% and 67.7% at 100 *μ*M, respectively.

The activity of compounds against promastigotes and amastigotes can differ, depending on the targets of antileishmanial action, which may be selective for one of two developmental forms. Overall, promastigotes may be more sensitive than intracellular amastigotes, because amastigotes are adapted to survive in hostile intracellular environment as well as due to the fact the compounds have direct contact with promastigotes; in contrast, to have anti-amastigote activity, substance must be capable of crossing the membrane of the host cell.

Moreover, unfortunately, the concentration required to give 50% inhibition of the growth of* L. amazonensis* (IC_50_) cannot be determined in the tests for evaluation of* in vitro* leishmanicidal activity because the plants extracts employed did not induce concentration-dependent killing. This effect can probably be a result of the large amount of substances present in the extracts of plants, which may have synergistic or antagonistic effects.

**Figure 3:**
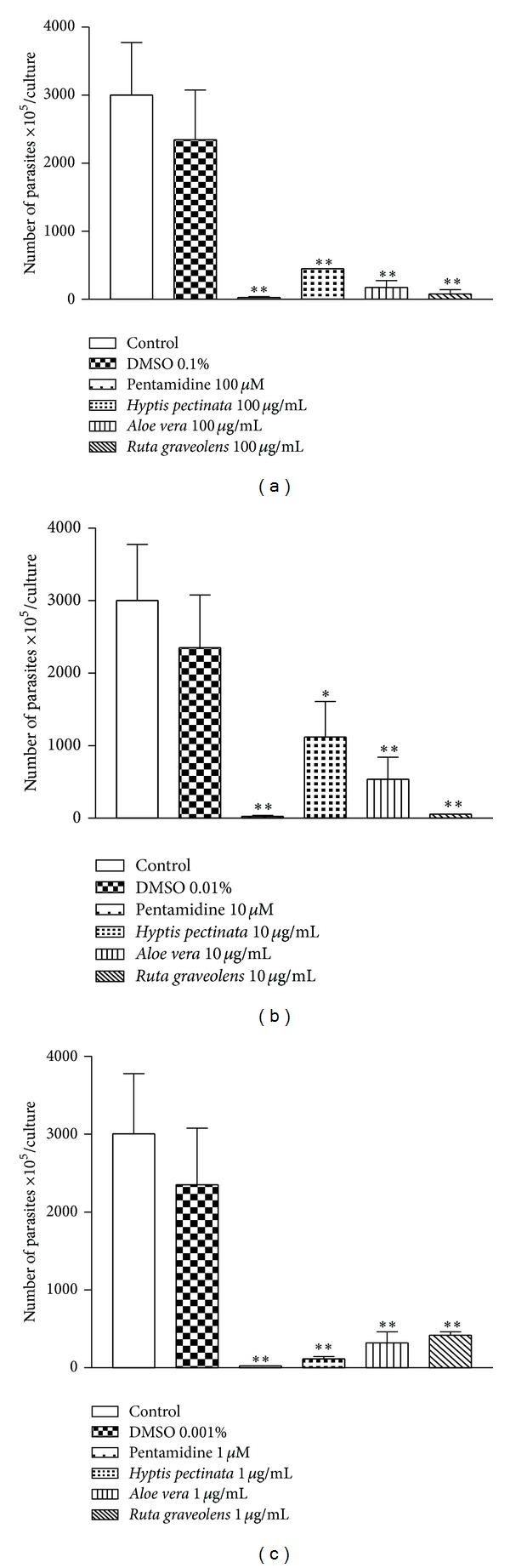
Antileishmanial effect of* H. pectinata*,* A. vera,* and* R. graveolens* against the replication of* L. amazonensis* promastigotes. The height of the bars indicates the parasite number at each concentration of plant aqueous extract compared with the control experiment containing only the solvent DMSO. Macrophages were infected with* L. amazonensis* promastigotes for 3 h. After infection, plant aqueous extracts were added at final concentrations of 100, 10, and 1 *μ*g/mL for 4 days. After 4 days, the extracellular load of* L. amazonensis* was measured. The experiments were performed three times independently, and each count was performed in triplicate. The data are reported as the means ± S.E.M. Differences with *P* values <0.05 were considered significant (*P < 0.05; **P < 0.01).

**Figure 4:**
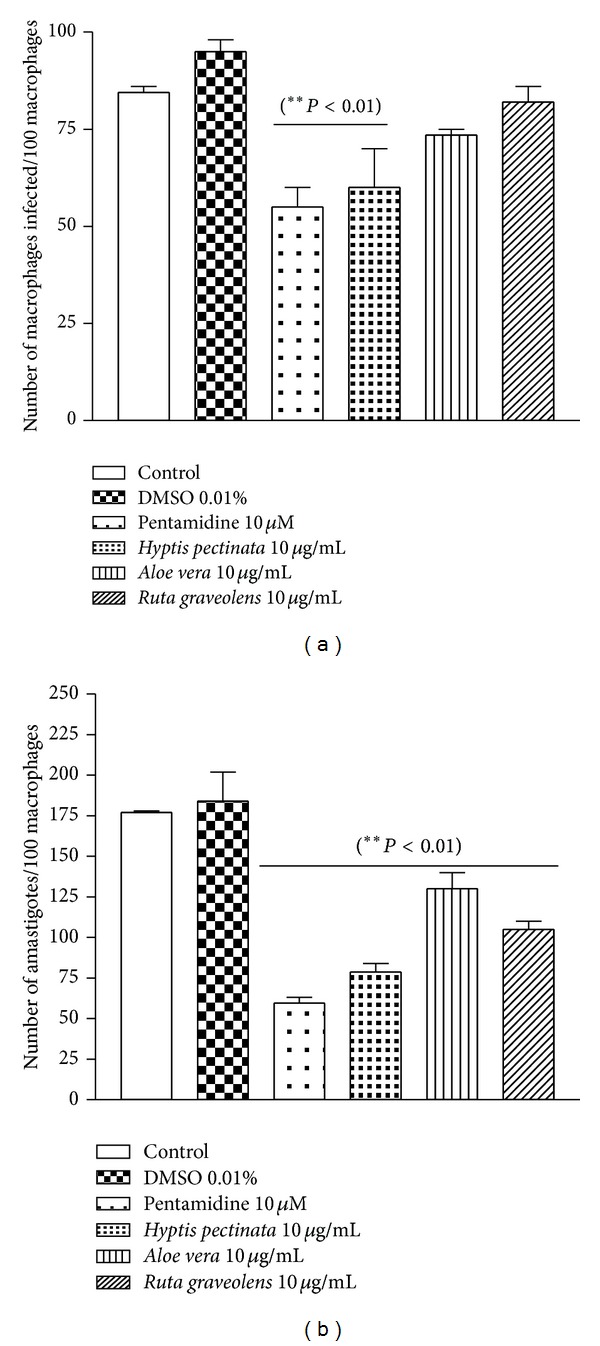
Antileishmanial effect of* H. pectinata*,* A. vera,* and* R. graveolens* against* L. amazonensis *amastigotes and the infection of macrophages. The height of the bars indicates the parasite number at each concentration of plant aqueous extract compared with the control experiment containing only the solvent DMSO. The numbers of infected macrophages (a) and intracellular amastigotes (b) were evaluated after the 3-day incubation with the plant extracts (10 *μ*g/mL). Macrophages were infected with* L. amazonensis* promastigotes for 3 h. After infection, the plant aqueous extracts were added at a final concentration of 10 *μ*g/mL for 3 days. Coverslips were washed, fixed, and stained, and the percentage of infected macrophages, noninfected macrophages, and intracellular amastigotes forms was determined by counting at least 100 cells/coverslip in triplicate on glass coverslips. Data are reported as the means ± S.E.M. Differences with *P* values <0.05 were considered significant.

*H. pectinata* L. is known popularly in Brazil as sambacaitá and is used to treat inflammation, bacterial infections, and ache [25]. This plant exhibits antibacterial activity [26] and antinociceptive and antiedematogenic action, with low toxicity reported in the literature [27]. This study expands the scientific knowledge of this plant by demonstrating its promising activity against* L. amazonensis*. This plant inhibited promastigote growth by 81.9% at 100 *μ*g/mL (Figure 3) and inhibited amastigote growth by 55.3% at 10 *μ*g/mL (Figure 4), both with *P* < 0.01.

*H. pectinata* presents significant amounts ofhyptolide and pectinolides A–C, among other 6-substituted-5,6-dihydro-*α*-pyrones [28] and pyrones exhibit antileishmanial activity [29]. Moreover, two pyrones isolated from the seeds of* Podolepis hieracioides *exhibited* in vitro* leishmanicidal activity against the promastigote forms of* L. donovani*,* L. major*,* L. infantum,* and* L. enriettii* and against the intracellular amastigote form of* L. donovani* [30]. The well-described constituents of* H. pectinata *include phytochemicals such as some terpenes, and its antileishmanial activity may be due to the presence of sesquiterpenes, such as *β*-caryophyllene and caryophyllene oxide, and to other terpenes that possess antimicrobial properties [26]. The chemical study of leaves from* Hyptis pectinata* resulted in the isolation of two new compounds, sambacaitaric acid (1) and 3-O-methyl-sambacaitaric acid (2), and nine known compounds, rosmarinic acid (3), 3-O-methyl-rosmarinic acid (4), ethyl caffeate (5), nepetoidin A (6), nepetoidin B (7), cirsiliol (8), cirsimaritin (9), 7-O-methylluteolin (10), and genkwanin (11). The EtOH extract, the hexane, EtOAc, and MeOH:H_2_O fractions, and compounds 1, 2, and 4 exhibited antileishmanial activity [31].

Previous work using* A. vera* leaf exudate demonstrated its potential against promastigotes of* L. mexicana*,* L. tropica*,* L. braziliensis*,* L. major,* and* L. infantum* and against the axenic amastigotes of* L. donovani* [14]. Such studies are in agreement with the results observed here, which demonstrated strong* in vitro* leishmanicidal activity against promastigotes (82.9% growth inhibition at 100 *μ*g/mL) and amastigotes (26.1% growth inhibition at 10 *μ*g/mL), as such shown in Figures 3 and 4, respectively. The presence of alkaloids, triterpenes, cyanidins, proanthocyanidins, tannins, and saponins in* A. vera* leaf exudate was demonstrated. Alkaloids, triterpenes, and saponin-like compounds individually and synergistically have leishmanicidal activity [23].

Dutta et al. [32] demonstrated that the incubation of promastigotes with* A. vera* leaf exudate causes promastigote death through an apoptosis-like mechanism similar to metazoan apoptosis. However, the pathways of induction and/or execution differed at the molecular level because the* A. vera* leaf exudate-induced leishmanicidal effect did not involve caspases and major proteases, an increase in cytosolic Ca^2+^, or the generation of reactive oxygen species. Additionally,* A. vera* leaf exudate also increases nitric oxide production. Nitric oxide causes extensive fragmentation of nuclear DNA in both axenic and intracellular amastigotes of* L. amazonensis*, and this fragmentation signal is regulated by non-caspase proteases of the proteasome [23].

*R. graveolens *(commonly known as rue) has been considered a medicinal plant since ancient times and is currently used to treat various disorders, such as aching pain, eye problems, rheumatism, and dermatitis [33]. In this study,* R. graveolens *significantly diminished the number of promastigotes presenting percentages of inhibition of 74.4% at 100 *μ*g/mL (Figure 3) and exhibited anti-amastigote activity of 40.3% at 10 *μ*g/mL (Figure 4).

Although pharmacological data regarding the antileishmanial activity of* R. graveolens* has not been documented in the literature, some chemical constituents of this plant, such as rutacridone, gravacridonediol, and rhodesiacridone, possess antileishmanial activity [34]. The mechanism of action of some alkaloids has been attributed to their ability to intercalate DNA. In addition to these acridone alkaloids,* R. graveolens *is rich in coumarins [33, 35], chemical constituents that have been studied with respect to their cytotoxic activities against* Leishmania*; for example, coumarins of the mammea type have been purified from* Calophyllum brasiliense* and exhibit antileishmanial activities against* L. amazonensis* [36] and* L. braziliensis *[37].

The genus* Pfaffia* is a member of the Amaranthaceae family, and many of its Brazilian species have been commercialized as Brazilian ginseng; these species are used to treat diabetes and rheumatism and are used as a tonic and aphrodisiac.* P. glomerata* roots possess an* in vitro* cytotoxic effect against strains of* L. braziliensis*. A hydroalcoholic extract of* P. glomerata* is somewhat active against the promastigote forms of* L. braziliensis* (IC_50_ 168.6 *μ*g/mL) [38]. In the present study,* P. glomerata* exhibited direct activity against extracellular forms of the parasite, inhibiting their growth by 88.7% at 100 *μ*g/mL (Figure 3). In addition, the mechanism of the antileishmanial action of this plant might involve the enhanced production of nitric oxide becauseitwas able to protect the gastric mucosa by increasing the concentrations of nitric oxide in the stomach [39].

*C. ambrosioides* has been used to treat cutaneous leishmaniasis due to* L. braziliensis* among the rural population of a cocoa-producing coastal area of Bahia state, Brazil [40]. In this study,* C. ambrosioides* exhibited direct activity against extracellular forms of the parasite, inhibiting growth by 87.4% at 100 *μ*g/mL (Figure 3).

*C. ambrosioides* also exhibited* in vitro* leishmanicidal effects against* L. amazonensis* promastigotes [41]. In the same way, Monzote et al. [42] showed that an essential oil from* C. ambrosioides* inhibits the progression of leishmanial infection both* in vitro* and* in vivo*. The essential oil had a minimal inhibitoryconcentration and EC_50_ values of 27.82 and 3.74 *μ*g/mL, respectively, against promastigotes of* L. amazonensis* [43]. Moreover, Patrício et al. [44] suggested that treatment with hydroalcoholic extracts of this plant by the intralesional route not only affects the regulatory mechanisms that control the dissemination of* L. amazonensis* but also appears to have a direct leishmanicidal effect. Previous work demonstrated that* C. ambrosioides* has the ability to recruit macrophages and further affects macrophage activation, which is fundamental to the control of* Leishmania*, by inducing nitric oxide [45]. The exact mechanism of action behind the direct antileishmanial effect exhibited by the essential oil of* C. ambrosioides* is unknown, but some authors have hypothesized that ascaridole (endoperoxide), an active molecule, generates free radicals that act on parasitic DNA [43]. Some authors have reported the medical use of these plants in the northeast of Brazil against intestinal parasites [46, 47].

The results obtained here represent a worthwhile characterization of the antileishmanial activity of extracts from traditional medicinal plants from the Brazilian flora.

## 4. Conclusion

The five species of plants that are traditionally used to treat cutaneous leishmaniasis were active against promastigotes, and* H. pectinata*,* A. vera,* and* R. graveolens* were active against intracellular amastigotes of* L. amazonensis*. To our knowledge, these data corroborate, for the first time, the ethnopharmacological use of these plants for the treatment of Leishmaniasis.
